# Interferometric measurements of refractive index and dispersion at high pressure

**DOI:** 10.1038/s41598-021-84883-6

**Published:** 2021-03-10

**Authors:** Yong-Jae Kim, Peter M. Celliers, Jon H. Eggert, Amy Lazicki, Marius Millot

**Affiliations:** Lawrence Livermore National Laboratory, Livermore, CA 94550 USA

**Keywords:** Optical spectroscopy, Electronic properties and materials

## Abstract

We describe a high precision interferometer system to measure the pressure dependence of the refractive index and its dispersion in the diamond anvil cell (DAC). The reflective Fabry–Perot fringe patterns created by both a white light and a monochromatic beam are recorded to determine both the sample thickness and its index at the laser wavelength and to characterize the dispersion in the visible range. Advances in sample preparation, optical setup, and data analysis enable us to achieve $10^{-4}$ random uncertainty, demonstrated with an air sample, a factor of five improvement over the best previous DAC measurement. New data on ${\text{H}}_{2}\text{O}$ liquid water and ice VI up to 2.21 GPa at room temperature illustrate how higher precision measurements of the index and its optical dispersion open up new opportunities to reveal subtle changes in the electronic structure of water at high pressure.

## Introduction

Refraction is one of the most common optical phenomena, indicating the bending of non-perpendicular incident waves at the interface between two different media. It reflects the dynamic polarization of atoms and/or molecules under electromagnetic radiation inducing oscillations of electron clouds and/or rotations of polar molecules^1,2^. By definition, the refractive index *n* determines the phase velocity of the electromagnetic wave in a given material *v* compared to the speed of light in vacuum as $n = c/v$. Empirically, it has been found to correlate strongly with other material properties such as density and polarizability^3–6^. In electro-magnetic theory, the complex refractive index can be defined as the square root of the complex relative dielectric constant^1,2^. The variations of the refractive index with photon energy, called the dispersion, can be used to reveal electronic properties such as the band gap energy^7–9^.

Measuring the refractive index accurately is therefore a powerful, contact-less way to probe how pressure alters the electronic charge distribution and the inter-atomic/molecular distances. Refractive index at high pressure has been extensively investigated using diamond anvil cells (DACs)^9–28^. With known refractive index, sample thickness in the DAC can be estimated for the further evaluations of the volumetric strain and equation of state (EOS) as well as for the detection of phase transitions^12,22^. One can also use the measured pressure dependence of the refractive index to estimate the density change using the Lorentz–Lorenz relation with the assumption of a constant molecular polarizability^27^. Compression-induced electronic transitions, like band-gap closure and metallization, have also been investigated by analyzing the dispersion^15–17,23^. Brillouin spectroscopy measurements with 90° and 180° scattering geometries require the refractive index to calculate the sound velocity and elastic properties of sample from the raw data^27,29^. Finally, the refractive index of the compressed sample is crucially needed to extract the accurate shock EOS data in shock compression experiments using optical velocimetry^30–32^.

**Figure 1:**
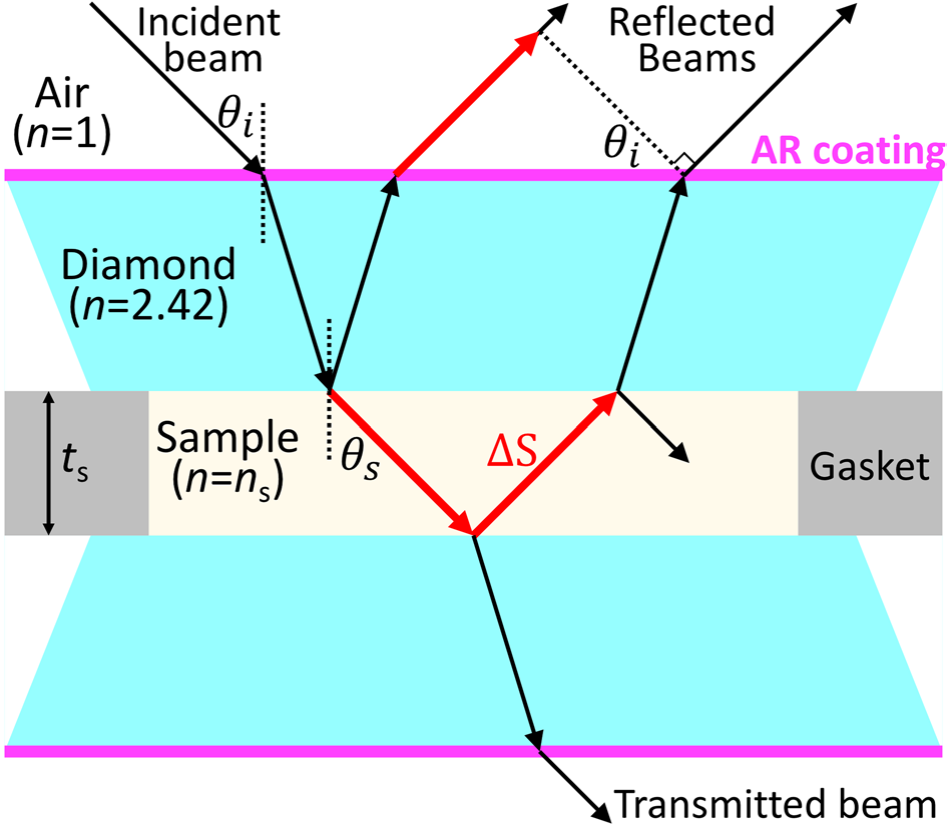
Schematic of the multiple reflections of an incident beam in the sample chamber acting as a Fabry–Perot cavity between the two diamond culet surfaces. Red arrows indicate the optical path difference ($\Delta S$) of the first two reflected beams from the DAC.

Fabry–Perot interferometry is an elegant way to measure the refractive index of a sample in the DAC. If the incident beam is reflected multiple times between two sample-diamond interfaces, the reflected (or transmitted) beams from the DAC interfere with each other to form a fringe pattern (Fig. 1). Writing the optical path difference, $\Delta S$, we can determine the phase difference, $\delta$, and the order of interference, *k*, as followed,

1a $$\begin{aligned} \Delta S&= 2n_{s}t_{s}\cos \theta _{s} = 2t_{s}\sqrt{n^{2}_{s}-n^{2}_{air}\sin ^{2}\theta _{i}}, \end{aligned}$$

1b $$\begin{aligned} \delta&= \frac{ 2\pi }{\lambda _{i} }\Delta S = \frac{ 4 \pi t_{s}}{\lambda _{i} }\sqrt{n^{2}_{s}-n^{2}_{air}\sin ^{2}\theta _{i}}, \end{aligned}$$

1c $$\begin{aligned} k&= \frac{\delta }{2\pi } = \frac{2t_{s}}{\lambda _{i}} \sqrt{n^{2}_{s}-n^{2}_{air}\sin ^{2}\theta _{i}} \nonumber \\&= \frac{2t_{s}}{\lambda _{i}} \sqrt{n^{2}_{s}-n^{2}_{air}\frac{r^{2}}{A^{2}+r^{2}}}, \end{aligned}$$

where $n_{s}$ and $t_{s}$ are the refractive index and thickness of the sample, $n_{air}$ is the refractive index of air (1.000278 at the laser wavelength $\lambda _L$ of 532 nm^33^), $\theta _{i}$ and $\theta _{s}$ are the incident and refractive angles of the beam which are related by Snell’s law, $\lambda _{i}$ is the wavelength of the incident beam, and *A* is the geometric calibration constant ($A = r/\tan \theta _{i}$) which is related to the working distance of the objective lens and allows us to use the radius of interference pattern (*r*) instead of its incident angle ($\theta _{i}$)^11^. According to Eq. (1c), the refractive index of a sample in the DAC can be measured by analyzing the fringe patterns which are obtained from different sample thicknesses^9^ or incident angles^22^ at a known wavelength. The intensity of the fringe pattern at a fixed thickness and angle can also be used to calculate the reflectivity at the sample-diamond interface and the refractive index of the sample^17,25,34^. However, experimental constraints have limited the measurement precision. As examples, a spacer plate inside the sample chamber provides a thickness difference but, at the same time, makes the fringe pattern much more complicated^9^. The rotation of the DAC is limited to only a few degrees^22^. Sample thickness in the DAC cannot be exactly estimated due to the misalignment and cupping of the anvils^9,22^. And, since the refractive index of the diamond anvil does not change much with moderate pressure^24^, if the diamond culets are not coated, the fringe contrast decreases significantly with increasing sample index during compression. This contrast loss can be utilized in the refractive index matching technique^12,21^, but this simple method provides at most two refractive index values in the typical DAC.

In this study, we built a Fabry–Perot interferometer setup to measure the refractive index of a transparent sample in the DAC, building on previous work by Le Toullec et al.^10–12^. They used a parallel white light and a converging monochromatic beam to probe the refractive index and thickness of a sample in the DAC independently at a single sample location. Our method has advantages for overcoming the issues in other Fabry–Perot techniques described above. Several improvements in the experimental setup and the data analysis methodology (such as anti-reflection coatings on the diamonds, the use of an objective lens with a high numerical aperture, and a sinusoidal fitting of the fringe spectrum after a Hilbert transform) allow us to reach the random uncertainty as good as $10^{-4}$ in the refractive index and its dispersion, which is demonstrated with an air sample in the empty DAC. We illustrate the use of our advanced interferometry technique by examining the refractive index and dispersion of water at high pressure and revealing the subtle change in its electronic structure. Finally, we discuss the possible sources of random and systematic uncertainties in this experiment.

In addition to the optical properties and the electronic structure, this technique can be further extended to study the polarizability, the equation of state (EOS) and the phase diagram of pure materials as well as their mixtures at high pressure. For example^35^, reported the mixing of water and methane ($\text{CH}_{4}$) at high pressure which are immiscible at ambient condition due to their polar and non-polar characters. As water and methane are expected to be among the main constituent materials of icy planets such as Uranus and Neptune, this interferometry technique can provide information on the structure and differentiation deep inside the planets.

**Figure 2:**
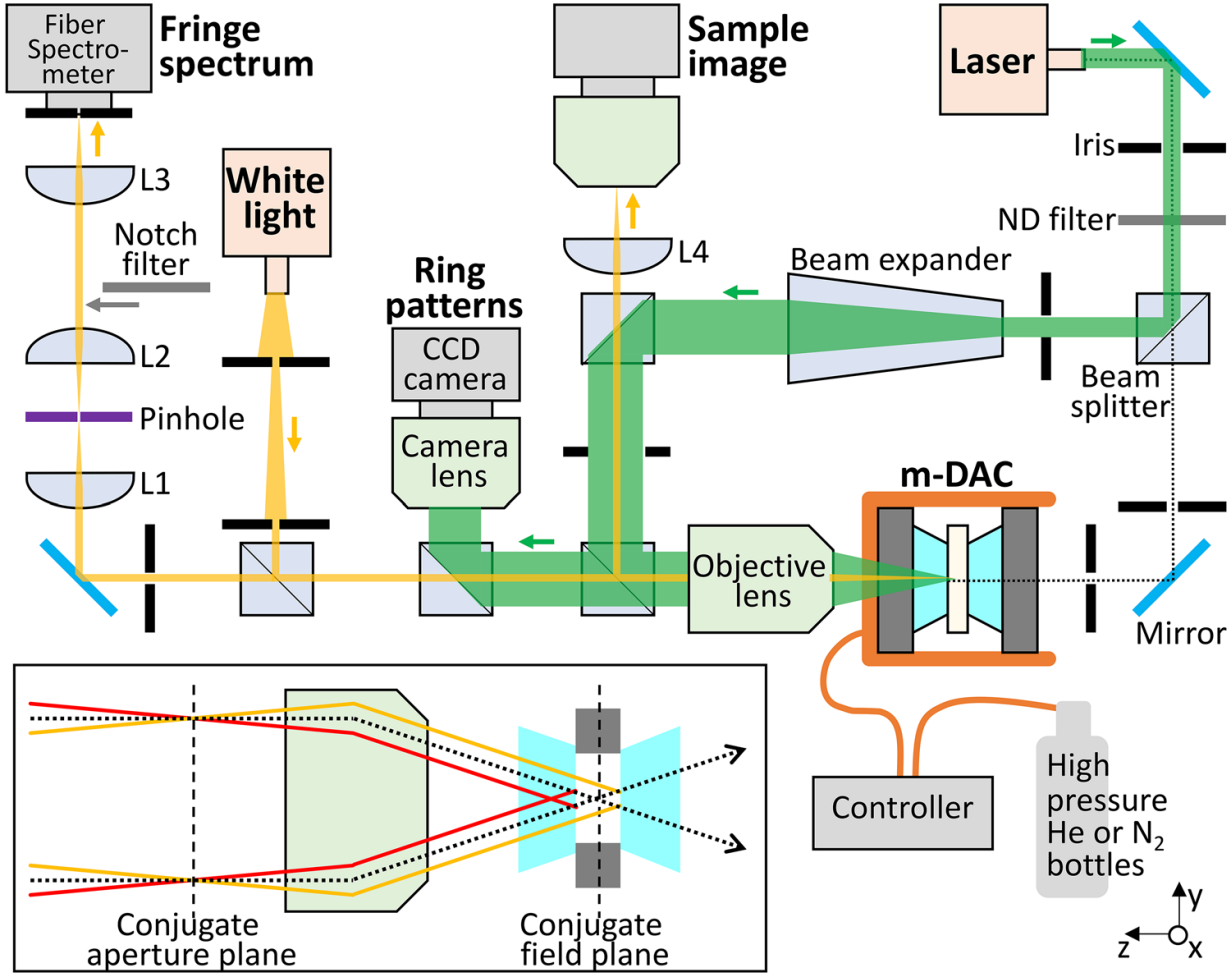
Schematic of our experimental setup. Interference fringe spectrum and ring pattern are obtained from the sample in the DAC with white (yellow) and laser (green) beams, respectively. Arrows indicate the direction of beam propagation from light sources to the sample through the objective lens and to the camera or the spectrometer. We also use a laser to align the tilt and rotation angles of the DAC (black dotted). The focal lengths of the achromatic doublet (*L*1 and *L*2) and plano-convex (*L*3 and *L*4) lenses are 50, 50, 100, and 200 mm, respectively. A 300 µm diameter pinhole is at the focus of *L*1. Bottom left inset shows the focuses of a parallel incident beam (black dotted) and two reflected beams (red and yellow) at the conjugate focal planes (black dashed).

## Experiments

### Sample preparation

We used four-pin stainless-steel DACs to generate high pressure conditions in the GPa range. Two diamond anvils with a thickness of 1.2 mm were anti-reflection (AR) coated for 532 nm on their table surfaces and glued on tungsten carbide seats with epoxy. A 25 µm thick tungsten plate was drilled with a 500 µm diameter hole and placed on the 800 µm diameter culet surface of the bottom anvil to serve as a gasket and create a cylindrical sample chamber. Deionized water (Sigma-Aldrich) was loaded inside the sample chamber together with a 5–10 µm diameter ruby ball^36^, and pressure was applied by tightening compression screws. We note that using a high-pressure gas membrane would be beneficial since it allows one to increase the pressure without realigning the cell. Pressure was measured from the shift of the $R_{1}$ ruby fluorescence peak excited by a 532 nm laser^37^. We assumed negligible pressure gradient in the sample at the relatively low pressure level examined in this study (2.21 GPa).

### Optical setup

The interferometer used in this study consists of two parts using a white light source and a laser beam (Fig. 2). First, the initially divergent white-light (KL 2500 LED, Schott) is reduced by passing through two irises and focused at the middle of the sample in the DAC with an infinite conjugate, plan-apochromatic objective lens having a magnification of 50$\times$ and a numerical aperture (NA) of 0.55 (378-805-3, Mitutoyo). The sample region probed by the white light is $\sim$ 5 µm diameter. An imaging relay, including spatial filtering through a confocal pinhole to improve the signal-to-background ratio of the fringe spectrum, transports the reflected white light from the DAC to the entrance of a fiber-coupled spectrometer (HR 4000, Ocean Optics) having a 600 lines/mm grating, a 25 µm entrance slit, and a 0.75 nm resolution in 450–900 nm range. A CCD camera with a resolution of 2592 $\times$ 1044 pixels and a pixel size of 2 $\upmu \text{m}$ (a2A2590-60ucBAS, Basler) allows us to record snapshots of the sample chamber during the measurements with a field of view (FOV) of 94 $\times$ 70 $\upmu$ m$^{2}$ and a total magnification of 55$\times$. Note that the notch filter shown in Fig. 2 is inserted only for pressure measurements.

The sample is also illuminated with a monochromatic Gaussian laser beam with a wavelength ($\lambda _{L}$) of 532 nm (CPS532, Thorlabs). The laser is expanded to the pupil diameter of the objective lens (4.4 mm) by a beam expander (2–5 $\times$, BE02-05-A, Thorlabs) and inserted in the optical path of the white light using a 50/50 cube beam splitter to co-propagate onto the sample through the objective lens. The Gaussian beam is focused to a $\sim$ 1 µm spot ($w_{0}$) with an intrinsic divergence of $\sim$ 10° ($\lambda _{L}/\pi w_{0}$). An interference ring pattern is captured by an additional CCD camera at the conjugate aperture plane where two reflected beams from the sample-diamond interfaces are focused and the real image of the ring pattern is formed (see the inset of Fig. 2). To optimize the intensity of the patterns, we use neutral density (ND) filters to adjust the incident laser intensity.

### Measurement procedure

Prior to inserting the DAC, we carefully align the optical setup with the 532 nm laser source, making sure that the beam is centered on the optics and detectors and that the white and laser beams are co-aligned. The spectrometer dispersion is calibrated with a standard Ne lamp.

Once the DAC is loaded with a sample and inserted in the beam path, the faint laser reflection from the culet surface of the back anvil is utilized to align the DAC by adjusting the xyz position and the tilt and rotation angles of the DAC. Then, pressure is measured by focusing a laser spot onto a ruby ball and collecting the characteristic $R_{1}$ and $R_{2}$ luminescence pattern with the fiber-coupled spectrometer. During the ruby luminescence measurements, the laser intensity was reduced by ND filters to prevent a possible temperature rise in the ruby. Then, after making a focus at the middle of the sample (see section “Focus position”), we collected the monochromatic interference ring pattern image and then the white-light fringe spectrum. A reflection spectrum from the gasket surface was obtained to normalize the wavelength-dependent intensity of the light source in the white light fringe spectrum. As particles or boundaries can distort the interferograms, it is important to make the measurement in a clear, uniform region of the sample. This procedure is repeated after each pressure change and equilibration.

An additional reference measurement with a known refractive index is needed to determine the geometric calibration constant (*A*) in Eq. (1c). Here, an empty gasket filled with air is used as the reference. The empty cell is prepared before the sample loading in the DAC. We also measure this cell at the end of the high pressure run after removing the sample from the DAC. Although both cells yield almost identical *A* values within $\sim$ 0.1%, we usually adopt the latter one obtained with the thinner gasket. The experimental sequence for the reference is identical to the one described above: alignment, ring pattern, then fringe and reflection spectra.

### Data analysis

#### *Calibration constant from an empty DAC*

##### Interference order and sample thickness from fringe spectrum

**Figure 3:**
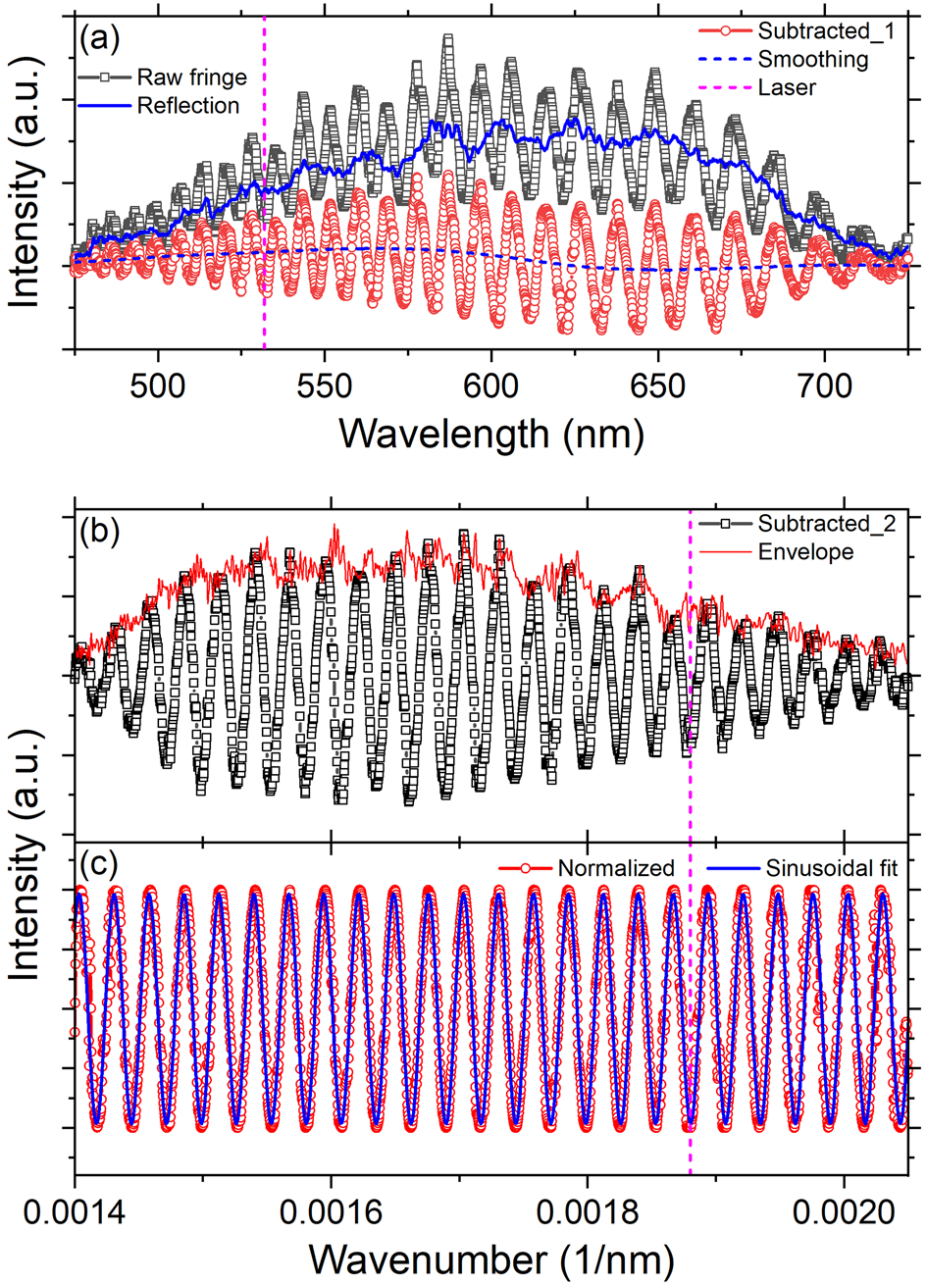
Analysis of the interference fringe spectrum obtained from the empty DAC with a white light. (**a**) The reflection spectrum from the flat gasket surface (blue line) is subtracted from the raw fringe spectrum (black square). In the subtracted spectrum (red circle), the residual intensity offset is determined with a smoothing method (blue dashed) and subtracted. (**b**) The envelope of the zero-mean spectrum (black square) is obtained with the Hilbert transform (red line). (**c**) The oscillation period ($\Delta \nu$) is obtained through a sinusoidal fitting (blue line) of the intensity-normalized spectrum (red circle). Pink dotted vertical line indicates the laser wavenumber ($\nu _{L}$).

The first step in the data analysis is to analyze the fringe spectrum obtained from the empty DAC. The reflected beams at the first diamond-sample and second sample-diamond interfaces are 180° out-of-phase. Therefore, intensity minima in the reflective interference spectrum satisfy the integral interference orders. By comparing two interference orders at adjacent peak minima (Supplementary Information S1), Eq. (1c) with a normal incidence ($\theta _{i}$ = 0°) can be re-written in terms of wavenumber ($\nu =\lambda ^{-1}$) as

2 $$\begin{aligned} \begin{aligned} k = 2n_{s}t_{s}\nu = \frac{\nu }{\Delta \nu }. \end{aligned} \end{aligned}$$

This equation implies that, once the oscillation period $\Delta \nu$ is determined from the fringe spectrum at a specific wavenumber, the values of $t_{s}$ and *k* can be calculated for the following analysis of the ring patterns. We note that Eq. (2) is only valid when the dispersion of the index is negligible, like in air, and $\Delta \nu$ is much smaller than $\nu$ (Supplementary Information S1).

Figure 3 shows in detail how $\Delta \nu$ is determined. From the raw spectrum, we first subtract the reflection spectrum obtained from the gasket surface (Fig. 3a). The residual intensity offset after the subtraction is removed by using a locally weighted scatterplot smoothing (LOWESS) method with a span of 5–8% of the data points. The intensity of the zero-mean spectrum is normalized by using an envelope generated with the Hilbert transform (Fig. 3b and Supplementary Information S2). Then, $\Delta \nu$ is determined using a sinusoidal fitting (Fig. 3c). As shown in Fig. 3c, the fitted curve shows an excellent match with our data despite the slight noise in the data. Since the dispersion of air is negligible (Fig. 5c), the fitting range between $\nu _{1}$ and $\nu _{2}$ is set to be as wide as possible to cover the entire range of the observed oscillation. The obtained oscillation period more precisely corresponds to the period at the mean of the fitting range; $\Delta \nu _{mean}$ at $\nu _{mean} = (\nu _{1} + \nu _{2})/2$. Based on Eq. (2), we calculate the sample thickness at $\nu _{mean}$, $t_{s} = 1 / (2n_{s}\Delta \nu _{mean})$, then the interference order at the laser wavenumber $\nu _{L}$ (= $\lambda ^{-1}_{L}$), $k_{L} = 2n_{s}t_{s} \nu _{L} = \nu _{L} / \Delta \nu _{mean}$. As an example shown in Fig. 3, $t_{s}$ and $k_{L}$ are calculated as 18.411 ± 0.002 µm and 69.228 ± 0.006, respectively.

##### Calibration constant from ring pattern

**Figure 4:**
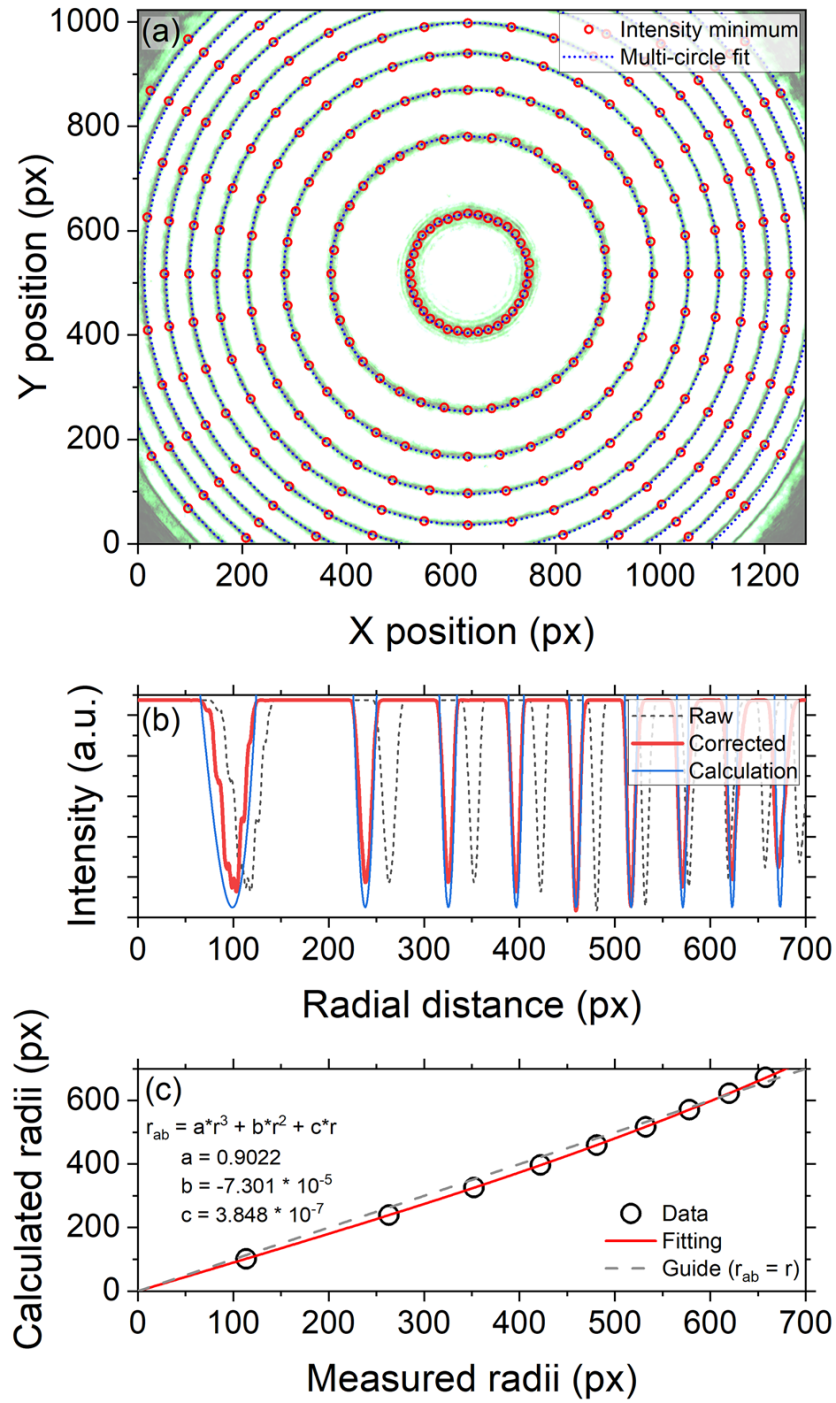
Analysis of the interference ring pattern obtained from the empty DAC ($t_{s}=$ 18.41 µm and $k_{L}=$ 69.23) with a 532 nm laser. (**a**) The intensity minima (red circle) are determined with a local Gaussian fitting of radial intensity profiles and fitted with a multiple-circle fitting method (blue dotted line) to determine the center and the radii of each ring. (**b**) The experimental (black dotted) and corrected (red) radial intensity profiles are compared with a calculated one (blue). Note that we overexpose the pattern in order to accentuate the locations of the minima and the amplitude of the calculated profile is properly adjusted to improve the visual comparison with the experimental profile. (**c**) Third-order polynomial fitting (red) between the experimental and calculated ring radii (black circle) with an identity line (gray dash).

The expanded laser beam converges onto the sample through the objective lens and the reflective interference ring pattern results from the succession of the destructive and constructive interferences as a function of the incident angle of the beam (Eq. 1c). Recording an image of the ring pattern allows us to readily obtain the radius of *m*-th ring, $r_{m}$, in pixel rather than its incident angle, $\theta _{i,m}$, where *m* is the order number of rings from the center (1, 2, 3, …). We can therefore determine the interference order of the *m*-th ring, $k_{m}$, which is an integer by using $k_{L}$ (i.e., *k* at $\theta _{i}=0$ or at the center of the pattern) and the calibration constant ($A = r / \tan \theta$) in Eq. (1c):

3 $$\begin{aligned} \begin{aligned} k_{m}&= [k_{L}] - m + 1 \\&= \frac{2t_{s}}{\lambda _{L}} \sqrt{n^{2}_{s}-\frac{r_{m}^{2}}{A^{2}+r^{2}_{m}}}, \end{aligned} \end{aligned}$$

where $[k_{L}]$ means the integer part of $k_{L}$. Then, using $t_{s}$ obtained above, Eq. (3) can be solved with the measurement of $r_{m}$ to calculate *A*.

The way to obtain $r_{m}$ and *A* is described as followed (Fig. 4a). The radial intensity profiles from a rough center position are extracted over 360° with a fixed angular binning (typically 10°). From each profile, intensity minima are determined with a local Gaussian fitting. Then, the exact center position and the ring radii, $r_{m}$, are obtained by using a multiple-circle fitting method on all intensity minima. Finally, the calibration constant, *A*, is determined by fitting a series of $k_{m}$ and $r_{m}$ values with Eq. (3). As an example, measuring ten ring patterns from the empty DAC in Figs. 3 and 4, we obtain *A* of 1254.9 ± 13.8 pixel.

#### Correction of image aberration

With all the known parameters in Eq. (3), the experimental interference pattern is compared with a theoretically calculated one. The intensity of the interference pattern in reflection mode, $I_{R}$, is expected to follow the Airy pattern^1^:

4 $$\begin{aligned} \begin{aligned} I_{R}&= \frac{ F\sin ^2(\frac{\delta }{2}) }{ 1+F\sin ^2(\frac{\delta }{2}) } I_{i}, \end{aligned} \end{aligned}$$

where $I_{i}$ is the incident beam intensity and the finesse *F* depends on the reflectivity *R* at the sample-diamond interface: $F = 4R / (1-R)^{2}$ with $R = | (n_{s}-n_{dia})/(n_{s}+n_{dia}) |^{2}$. The phase difference $\delta$ in Eq. (4) varies with the ring radius *r* and, from Eq. (1b), can be expressed in terms of the calibration constant *A* as $\delta = ( 4\pi t_{s} / \lambda _{L} )\sqrt{ n^{2}_{s} - r^{2}/( A^{2}+r^{2} ) }$.

As shown in Fig. 4b, the calculated radial intensity profile slightly deviates from the experimental one. This discrepancy is likely due to image aberrations, introduced, for example, by the use of the thick diamond anvils as the optical windows, their cupping and misalignment^38^, and the objective lens^12^ having a high numerical aperture (NA). To mitigate this issue, we empirically adopt a third-order polynomial fitting between the measured ring radii (*r*) and the calculated ones ($r_{ab}$) for the empty DAC using Eq. (3) as shown in Fig. 4c;

5 $$\begin{aligned} \begin{aligned} r_{ab} = ar^{3} + br^{2} + cr. \end{aligned} \end{aligned}$$

This image aberration correction relation (Eq. 5) is used to correct the experimental radial distance during the ring pattern analysis for the sample.

#### Refractive index of a compressed sample

Once the calibration constant *A* (Eq. 3) and the image aberration correction relation $r_{ab}$ (Eq. 5) are determined from the empty DAC, we are now ready to calculate the refractive index of the sample. The data analysis method is almost identical to the one discussed above for the empty DAC (see section “Calibration constant from an empty DAC”.

The white-light fringe spectrum from the sample is analyzed first. After the intensity normalization, the oscillation period $\Delta \nu _{mean}$ near $\nu _{mean}$ is obtained by a sinusoidal fitting. Then, $k_{L}$ at $\nu _{L}$ is calculated using $k_{L} = \nu _{L}/\Delta \nu _{mean}$. If the dispersion of the index in the sample is nontrivial, the value of $\nu /\Delta \nu$ in Eq. (2) is no longer equal to the interference order. This dispersion-induced error in determining *k* can be eliminated by using a thin sample (15–30 µm, in this study) and obtaining the interferograms when the first ring in the laser interference pattern is big enough (for more details, see section “Sample thickness” and Supplementary Information S1).

Then, in the interference ring pattern, the intensity minima are determined through a local Gaussian fitting. The ring radii $r_{m}$ are obtained using a multiple-circle fitting method and corrected for the image aberration $r_{m,ab}$ using Eq. (5). Finally, a series of $k_{m}$ and $r_{m,ab}$ are fitted with Eq. (3) to provide $n_{s}$ and $t_{s}$.

#### Dispersion of refractive index

**Figure 5:**
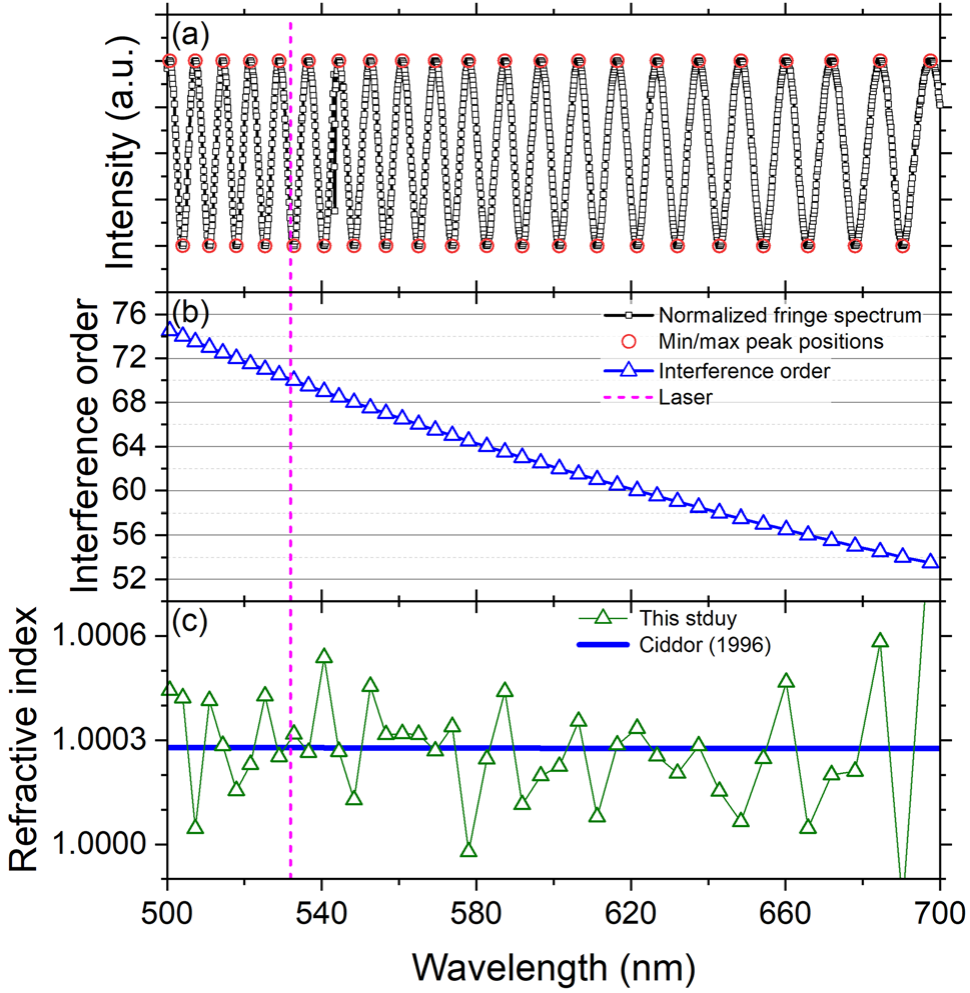
Dispersion of air. (**a**) Normalized spectrum (black square) in Fig. 3c with marks at the peaks (red circle). (**b**) Interference order at each peak (blue triangle) calculated with $k_{L}$ at $\lambda _{L}$ (pink dotted). (**c**) The obtained refractive index (green triangle) is compared with the literature data^33^ (thick blue line).

In addition to the determination of the refractive index at $\lambda _{L}$, our advanced setup allows us to evaluate the dispersion through further analysis of the white light fringe spectrum (Fig. 5). As explained in section “Calibration constant from an empty DAC”, the interference order, *k*, is an integer (or half-integer) at the peak minimum (or maximum). From $k_{L}$ at $\lambda _{L}$, we can trace the change of *k* at every peak (Fig. 5a,b). For example, with $k_{L}=69.23$ at 532 nm in Fig. 3, *k* = 69 and 69.5 at the minimum and maximum peaks near 532 nm. Finally, the refractive indices at the peaks can be calculated using Eq. (2) with the obtained $t_{s}$. The data points in Fig. 5c are scattered within ± 2 $\times$ 10$^{-4}$ which may be due to the uncertainty in determining peak positions and sample thickness, and follows well the expected trend from Ref.^33^ considering 15 °C, 101,325 Pa, and 0% humidity with 450 ppm CO$_{2}$.

## Results

### Refractive index of water

**Figure 6:**
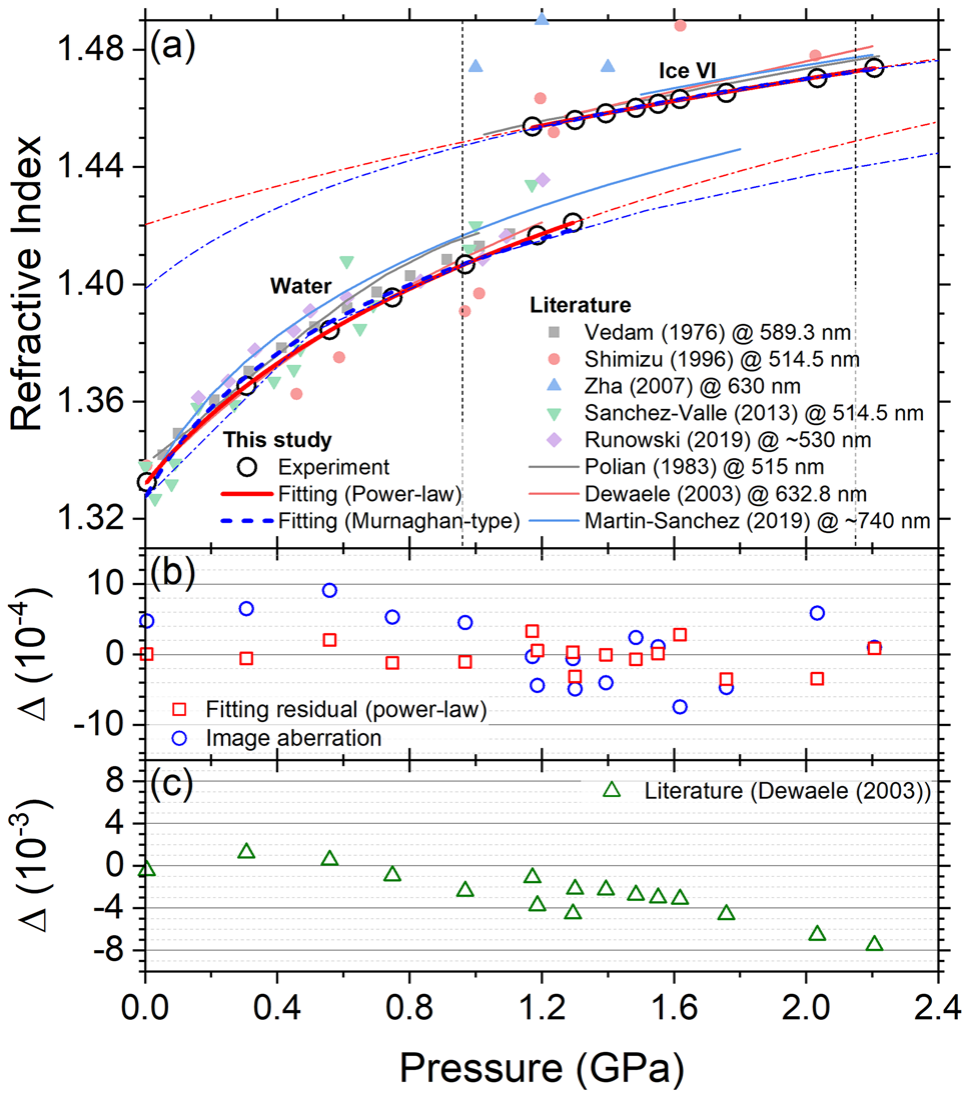
(**a**) Refractive index of $H_{2}O$ in liquid and ice VI phases measured at 532 nm in this study with literature values^12–14,17–20,39^. Our dataset is fitted with power-law^12^ and Murnaghan-type^19^ models (red solid and blue dashed lines) and the fitting curves are extrapolated to 0 and 2.4 GPa (thin red and blue dash-dotted lines). Equilibrium pressure levels for water-to-ice VI and ice VI-to-ice VII transitions (0.96 and 2.15 GPa at $25\,^\circ \hbox {C}$^40^) are indicated with black dotted vertical lines. Error for pressure is 0.03 GPa. Random and systematic errors for the index are 10$^{-4}$ and 5 $\times$ 10$^{-4}$, respectively. (**b**,**c**) The differences of our data from the power-law fitting curve (red square), the recalculated index using another correction relation for the image aberration $r'_{ab}$ (see section “Image aberration”, blue circle), and the literature result^12^ (green triangle).

**Figure 7:**
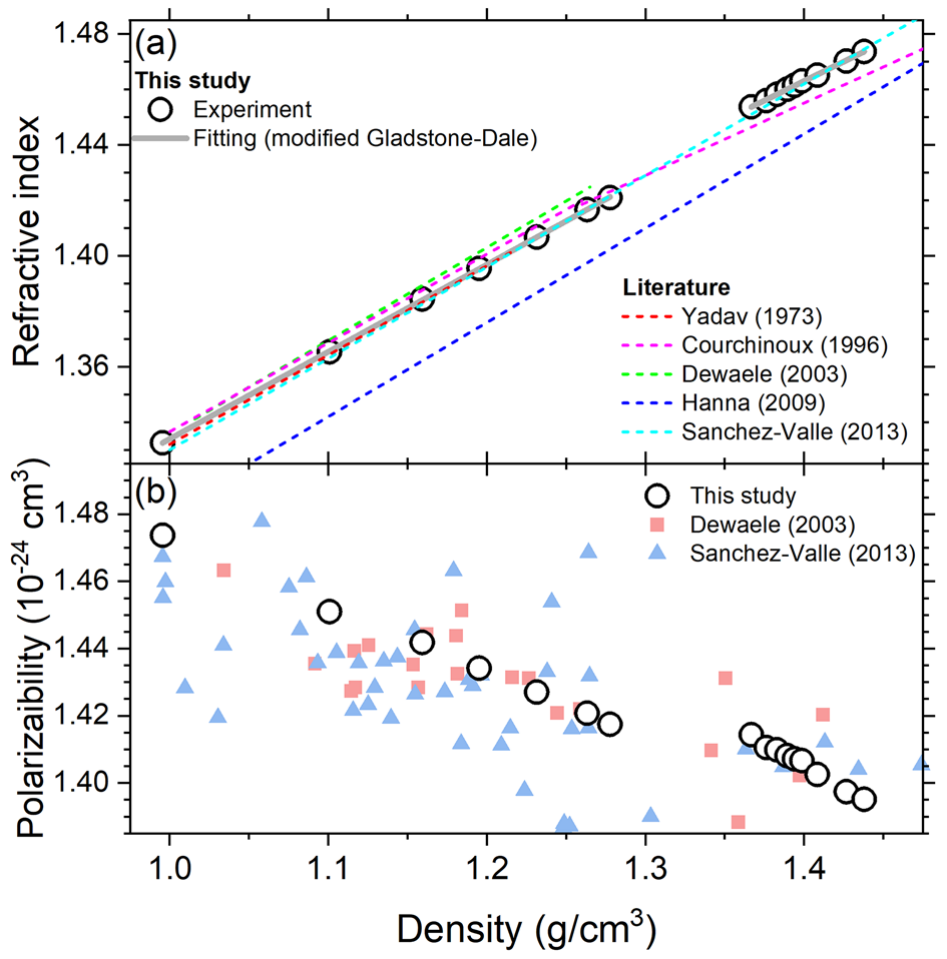
(**a**) Refractive index of water as a function of density. Our datasets in liquid and ice VI phases are fitted with a modified Gladstone–Dale model and compared with the model equations in the literatures^12,18,26,41,42^. (**b**) Molecular polarizability calculated using the Lorentz–Lorens model with literature values^12,18^.

To demonstrate the validity and accuracy of our interferometry measurements, the refractive index of water is examined up to 2.21 GPa at room temperature and compared to previous studies (Fig. 6a). As expected, the refractive index continuously increases with pressure in liquid water and ice VI with a sudden jump at 1.29 GPa. This discontinuity and the overlapped pressure range of their indices ($\sim$ 1.2 to 1.3 GPa) are due to the freezing of supercompressed liquid water at 1.29 GPa to ice VI causing a pressure drop to the equilibrium melting level of ice VI (0.96 GPa at $25\,^\circ \hbox {C}$^40^). One index is obtained also from supercompressed ice VI at 2.21 GPa slightly above the equilibrium transition pressure to ice VII (2.15 GPa^40^). The suppressed phase transition in the supercompressed phases is monitored by the camera during compression and evidenced by the continuity of the index with pressure (Fig. 6a) and density (Fig. 7a). Our dataset at 532 nm agrees well with previous studies^12–14,17–20,39^. The measured refractive index in liquid phase is almost overlapped with the index-pressure relation provided by Dewaele et al.^12^ and in a relatively good agreement with the results of Refs.^18,20,39^. In ice VI phase, our dataset is close to the results from previous studies ^12,13,19^, while the slope of our data is slightly lower. In Fig. 6c, we compare our dataset to the index-pressure relation provided by Ref.^12^, as our data are very close to their results and their measurement precision (or reproducibility) of 5 $\times$ 10$^{-4}$ is the best among other studies on water. The difference is less than 2 $\times$ 10$^{-3}$ in low-pressure liquid regime, but increases with pressure to 8 $\times$ 10$^{-3}$ in ice VI.

We find that our data for the increase of the refractive index with pressure can be well fitted with a power-law model^12^, $n = a_{1} + b_{1} \left( 1 + c_{1}P \right) ^{ d_{1} }$. Alternatively, we also provide a fit with a Murnaghan-type model^19^, $n = a_{2} \left[ 1 +(b_{2}/c_{2}) P \right] ^{1/b_{2}}$, that assumes a linear dependence of the index with density (like a modified Gladstone–Dale relation^4,12^) in the first-order Murnaghan pressure-volume EOS. We use as few parameters as possible to avoid parameter uncertainty and provide the comparison with previous studies; $a_{1}$ and $c_{2}$ are fixed as the values in the literature^12,19^. As our experimental dataset exhibits a clear trend in relatively narrow pressure range, both equations provide excellent fits. The fitting residual of a power-law model in liquid and ice VI phases has a standard deviation of $\sim$2 $\times$ 10$^{-4}$ (Fig. 6b), smaller than the best previous precision of 5 $\times$ 10$^{-4}$ (Ref.^12^). The residual of a Murnaghan-type model is $\sim$2 $\times$ 10$^{-3}$, less satisfactory particularly in water phase only due to a $c_{2}$ constraint. The fitting parameters and covariance matrices of both models to the pressure-dependent refractive index are summarized in Tables 1 and 2.

**Table 1:** Fitting parameters of power-law and Murnaghan-type models for the refractive index of water (0.05–1.29 GPa) and ice VI (1.17–2.21 GPa).

Phase	$a_{1}$ (fixed)	$b_{1}$	$c_{1}$	$d_{1}$	$a_{2}$	$b_{2}$	$c_{2}$ (fixed)
Water	0.900	0.432 ± 0.0001	2.653 ± 0.044	0.126 ± 0.001	1.327 ± 0.002	30.182 ± 2.036	6
Ice VI	0.425	0.995 ± 0.008	0.555 ± 0.479	0.065 ± 0.029	1.398 ± 0.0009	36.607 ± 0.961	14

The subscripts 1 and 2 refer to power-law and Murnaghan-type models, respectively.

**Table 2:** Covariance matrix elements for the power-law and Murnaghan-type fittings given in Table 1.

Phase	$\sigma _{b_{1}b_{1}}$	$\sigma _{b_{1}c_{1}}$	$\sigma _{b_{1}d_{1}}$	$\sigma _{c_{1}c_{1}}$	$\sigma _{c_{1}d_{1}}$	$\sigma _{d_{1}d_{1}}$	$\sigma _{a_{2}a_{2}}$	$\sigma _{a_{2}b_{2}}$	$\sigma _{b_{2}b_{2}}$
Water	$1.938 \times 10^{-8}$	$-3.646 \times 10^{-6}$	$6.139 \times 10^{-8}$	$1.915 \times 10^{-3}$	$-4.453 \times 10^{-5}$	$1.491 \times 10^{-6}$	$5.707\times 10^{-6}$	$4.287 \times 10^{-3}$	4.145
Ice VI	$6.143\times 10^{-5}$	$-3.724\times 10^{-3}$	$2.246 \times 10^{-4}$	$2.283 \times 10^{-1}$	$-1.383 \times 10^{-2}$	$8.394 \times 10^{-4}$	$7.400\times 10^{-7}$	$8.177 \times 10^{-4}$	0.923

**Table 3:** Fitting parameters and covariance matrix elements of the modified Gladstone–Dale model for water (0.05–1.29 GPa) and ice VI (1.17–2.21 GPa).

Phase	*a*	*b*	$\sigma _{aa}$	$\sigma _{ab}$	$\sigma _{bb}$
Water	1.020 ± 0.001	0.314 ± 0.001	$1.632 \times 10^{-6}$	$-1.381\times 10^{-6}$	$1.175 \times 10^{-6}$
Ice VI	1.064 ± 0.010	0.285 ± 0.007	$9.451 \times 10^{-5}$	$-6.751 \times 10^{-5}$	$4.824 \times 10^{-5}$

### Gladstone–Dale relation

The refractive index is plotted again in terms of density, $\rho$, in Fig. 7a using the equation of states of liquid water^43^ and ice VI^44^.This relation is often described with a modified version of the Gladstone–Dale model^3,4,12^;

6 $$\begin{aligned} n = a + b \rho . \end{aligned}$$

Our data are well fitted to this linear model with a fitting residual of $\sim$ 2 $\times$ 10$^{-4}$ but with slightly different slopes in each phase. In liquid water, our data match well the results by Refs.^18,41^ and are close to those by Refs.^12,42^. Our ice VI data still have a good agreement with the result by Refs.^18^, although their high density data near 1.4 g/cm$^{3}$ were obtained from liquid phase at a higher temperature (293–673 K) and pressure (0–7.1 GPa) regime. Further studies are required to determine if the refractive index is the sole function of density or is indeed affected by both external pressure-temperature condition and microstructure. For example, Courchinoux and Lalle^42^, provided two linear relations below and above 1.26 g/cm$^{3}$, and Dewaele et al.^12^ gave their best fits of the modified Gladstone–Dale model for liquid and ice VII phases separately. Otherwise, the relation of liquid water by Sanchez-Valle et al.^18^ is very close to our ice VI data, and Hanna and McCluskey^26^ provided one model fit for liquid, ice VI and ice VII. The fitting parameters and covariance matrices of this model are summarized in Table. 3.

### Application to shock wave velocimetry in water

The obtained index-pressure and index-density relations [*n*(*P*) and $n(\rho )$ in Tables 1 and 3] can be utilized for correcting optical velocimetry measurement during shock compression experiments. When a shock wave propagates through an initially transparent sample and the interface between the shocked and un-shocked sample is reflective, a measured, apparent shock velocity ($U_{s,app}$) can be corrected using the refractive index of the initial sample which is precompressed in a DAC or at ambient pressure;

7 $$\begin{aligned} \begin{aligned} U_{s}&=U_{s,app}/n_{0}, \end{aligned} \end{aligned}$$

where $U_{s}$ is the true shock velocity and $n_{0}$ is the refractive index at a initial sample pressure $n(P_{0})$ or density $n(\rho _{0})$^30–32^. Instead, if an interface behind the shock front is viewed through a transparent, shocked sample or window, an apparent particle velocity ($u_{p,app}$) is measured. Then, the true particle velocity can be obtained as

8 $$\begin{aligned} \begin{aligned} u_{p}&=u_{p,app}/a, \end{aligned} \end{aligned}$$

where *a* is *n* ($\rho =0$) or the zero-density intercept of the modified Gladstone–Dale model^30,45,46^.

### Molecular polarizability of water

The polarizability of water molecule can be extracted from the refractive index-density data through the Lorentz–Lorenz relation^1,5,6^,

9 $$\begin{aligned} \begin{aligned} R_{LL}&= \left( \frac{n^{2}-1}{n^{2}+2} \right) \rho ^{-1} = \left( \frac{4 \pi N_{A} }{3 M} \right) \alpha , \end{aligned} \end{aligned}$$

where $R_{LL}$ is the Lorentz–Lorens molar refractivity, *M* is the molar mass (18.015 g/mol for ${\text{H}}_{2}O$), $N_{A}$ is the Avogadro number ($6.0221 \times 10^{23}$ mol$^{-1}$), and $\alpha$ is the mean polarizability. Figure 7b shows that the polarizability (in Å$^{3}$) decreases almost linearly from 1.47 to 1.42 in the liquid and from 1.41 to 1.40 in ice VI. The corresponding Lorentz–Lorens molar refractivity (in cm$^{3}$/g) is 0.206–0.198 in the liquid and 0.198–0.195 in ice VI. Our values are consistent with the results of previous studies^12,18^ but are found to exhibit significantly less scatter. Such a decreasing polarizability with density can be understood as evidence for the reducing extent of the electronic cloud and the increasing confinement of the intermolecular interactions^12,18^. However, as the Lorentz–Lorenz model is only suitable for symmetric entities (e.g., gases which freely rotate and non-polar liquids)^12,47^ and assumes a point dipole without the overlap of electron distributions by the nearest neighbors^17^, further theoretical development would be needed to precisely determine the polarizability of compressed H$_{2}$O phases using refractive index measurements.

### Dispersion of water

**Figure 8:**
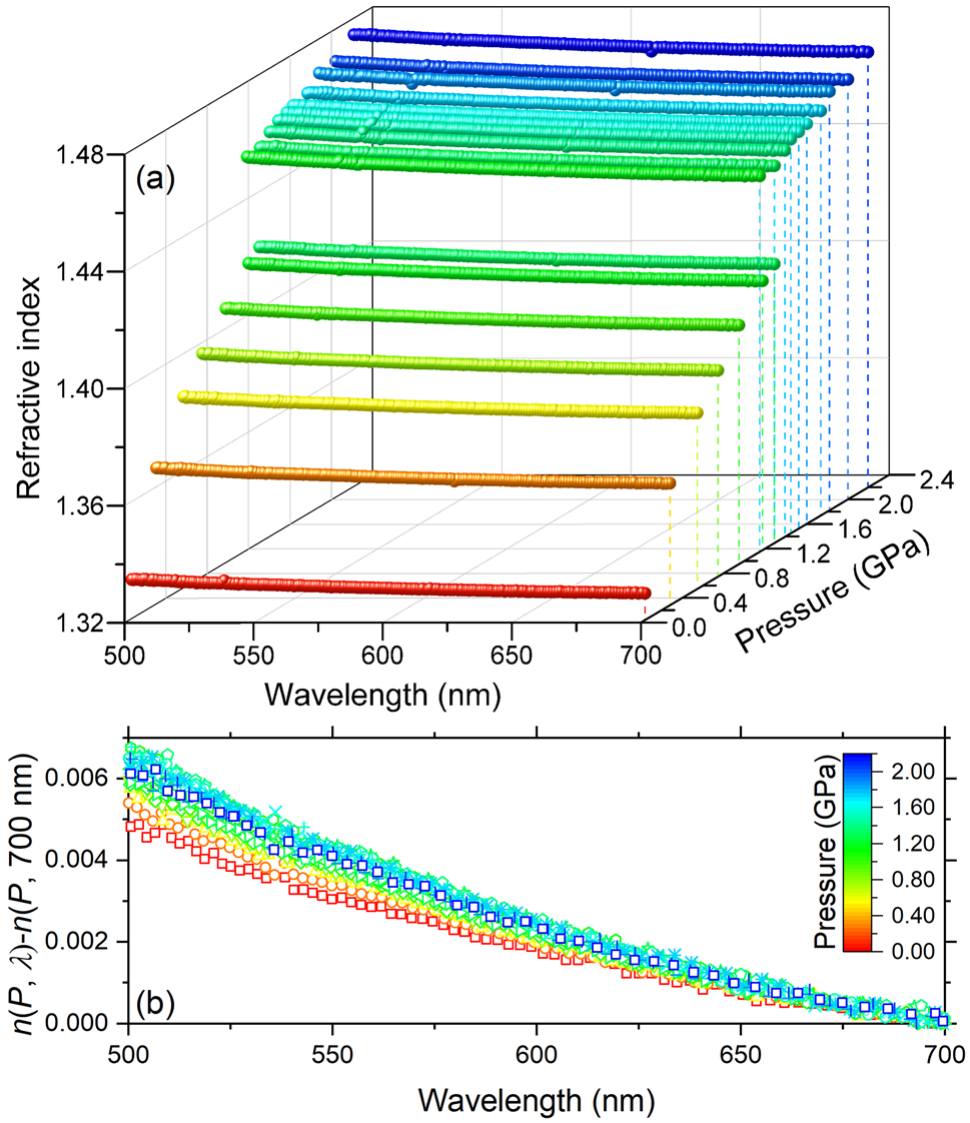
(**a**) 3-dimension plot of the refractive index in liquid water and ice VI as a function of wavelength and pressure. (**b**) The refractive index is normalized by subtracting the index at 700 nm to highlight the dispersion. The color scale represents the pressure levels on both panels (**a**,**b**).

The wavelength dependence of the refractive index of water with pressure is illustrated in Fig. 8a. A very subtle increase of the refractive index with decreasing wavelength (or increasing photon energy) is revealed: the dispersion is as small as 0.5% over 500–700 nm (or 2.48–1.77 eV) and 0.05–2.21 GPa ranges examined in this study (Fig. 8b). Previous study reported the absence of noticeable pressure effect on the dispersion up to 35.4 GPa^12^. However, we find that, with increasing pressure, the dispersion of the index (or $dn/d\lambda$) increases in liquid phase and remains almost constant. This discrepancy is possibly attributed to the small dispersion of water and the inherent difficulty of such measurements, often limited to relatively narrow wavelength ranges. Using the current improved setup and analysis techniques, we can now resolve subtle pressure-induced changes in the dispersion. The dispersion data can be used to infer the electronic structure of material^8,9,17,48^. A detail analysis of the dispersion and electronic properties such as the band gap up to a higher pressure level will be discussed in our forthcoming paper.

## Error analysis

In this section, we detail several important steps necessary to reach the 10$^{-4}$ precision in the refractive index that we report for the air sample.

### Coating of the diamonds

The first air-diamond interface is a possible source for a strong reflection due to the large difference of their refractive indices; $R = |( n_{dia} - n_{air} ) / ( n_{dia} + n_{air} ) |^{2} = 0.173$. Such a strong reflection induces detrimental distortion and degradation to the sample interferograms. In this study, we used anti-reflection coatings of 84 nm thick ${\rm Al}_{2}{\rm O}_{3}$ on the outer table surfaces of the anvils (Fig. 1). To explore a much higher pressure regime, additional partially-reflective coating on the inner culet surfaces^22^ may be desirable. Such coatings could help to prevent the contrast loss in the interferograms as the refractive index of sample approaches that of diamond and the anvil surfaces are cupped with increasing pressure.

### Sample thickness

The precise determination of *k* in the fringe spectrum is one of the most important requirements for a high precision measurement, as an error of ±1 in *k* brings a significant error of 1–10% in $n_{s}$. In a sample with a nontrivial dispersion, the value of $\nu /\Delta \nu$ (Eq. 2) is not equal to the interference order *k*. Their discrepancy increases with increasing *k* or, equivalently, $t_{s}$. Therefore, the use of a thin sample helps to avoid this dispersion-induced error in determining *k* (for more details, see Supplementary Information S1). Also, with a thin sample, we can minimize the error from wrong focus position (see section “Focus position”) and obtain the low-frequency fringe spectrum with sufficient data points per a single oscillation period for a better determination of $\Delta \nu$ through a sinusoidal fitting. In contrast, as $t_{s}$ decreases, the number of rings that can be observed within the numerical aperture of our optical system decreases, which affects the accuracy of our multiple-circle fitting procedure. In this study, the optimum value of $t_{s}$ is found as $\sim$15 to 30 µm, compromising a clear resolution in the fringe spectrum and a number of ring patterns which is at least 4.

### Alignment and spatial filtering

Non-uniform sample thickness inside the DAC is an inherent experimental limitation which also leads to difficulties in the EOS measurement^12,22^. As the interferogram is sensitive to the sample thickness, two independent white and laser beams are coincidently aligned to illuminate the same sample location. Also, the extension of the region probed by the white light beam is reduced as small as possible ($\sim$5 µm) using two irises near its source (Fig. 2), matching as much as possible the $\sim$1 µm focus spot of the laser beam.

### Lens choice

**Figure 9:**
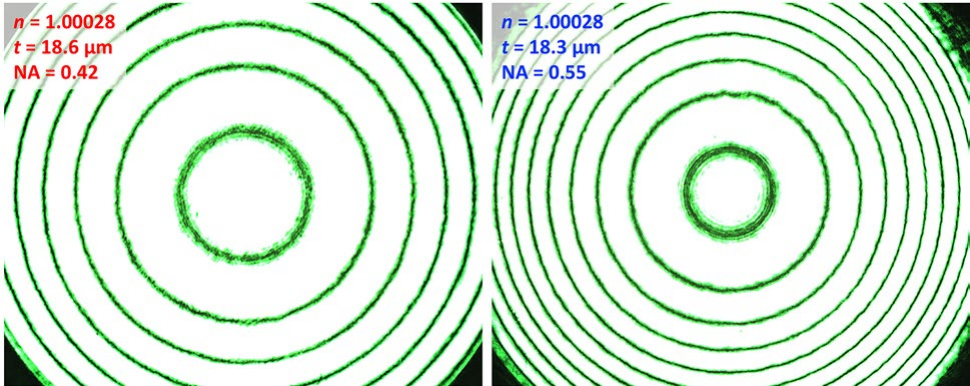
Comparison of ring patterns obtained from objective lenses having different numerical aperture (NA) values of (left) 0.42 and (right) 0.55. More rings can be obtained by a lens with a higher NA. The patterns are obtained from the empty DAC with an air gap.

The selection of a proper objective lens is critical for the ring pattern analysis. In addition to a working distance (WD) longer than the thickness of the upper half of the DAC, a high numerical aperture (NA) is required to preserve a wide incident angle and to collect multiple ring patterns, enhancing the accuracy of the multiple-circle fitting and of the $n_{s}$ and $t_{s}$ measurements. For example, when an objective lens with NA = 0.42 is replaced by that with NA = 0.55, the number of rings increases by 50% (Fig. 9). The use of high NA lens pays off especially at high pressure as both of sample thickness and ring pattern number diminish together, although it possibly increases the Image aberration (see section “Image aberration”).

### Focus position

**Figure 10:**
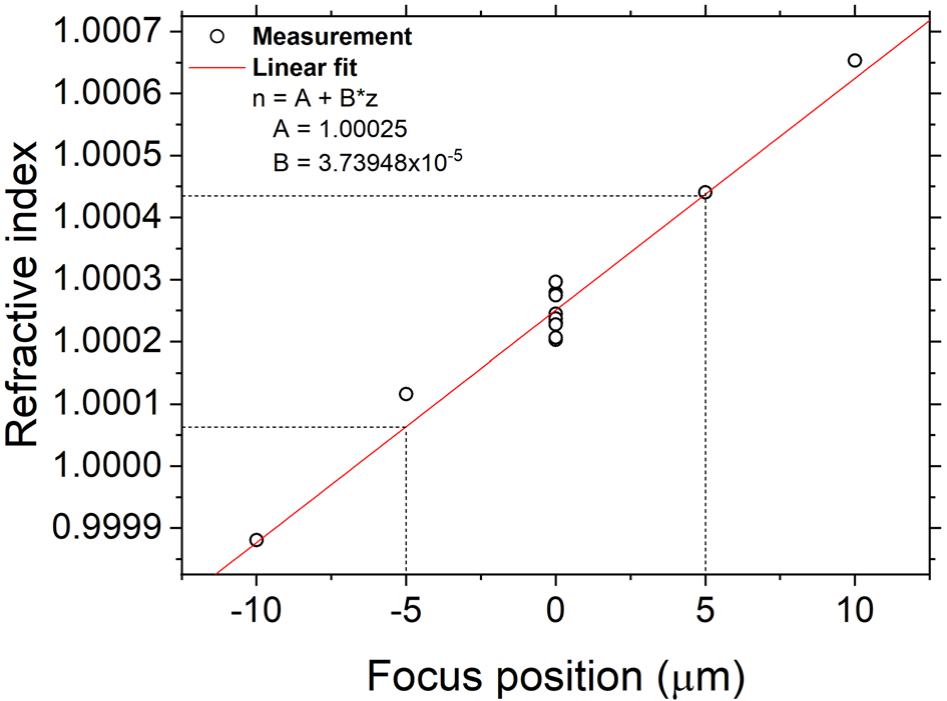
The inferred refractive index of air linearly varies with the focus position. The inaccuracy in the focus position possibly induces $$\sim$$$$10^{-4}$$ random error in the sample refractive index.

Once all the error sources mentioned above are resolved, the accuracy of our interferometry measurements is now mainly dependent on the focus position. Particularly, the focus position significantly affects the ring pattern analysis due to its angular resolution. The interferograms are taken at the middle of the sample thickness between the front- and rear-anvil culets. We first define the z positions of the two sample-anvil interfaces by manually adjusting the z-axis micrometer stage and focusing at the gasket surface and the dust particles on the culets, and then move the stage to their mid-position. The uncertainty related to the focus position is evaluated by measuring ten independent ring patterns from an empty DAC having a $\sim$18.4 µm gap. At each measurement, the focus position (in z axis) is re-adjusted while the sample location (in x and y axes) is fixed. The refractive indices from ten measurements at 0 µm (the middle of sample thickness) are scattered within $10^{-4}$ as shown in Fig. 10, which is our random measurement uncertainty. In addition, we find a linear increase of the refractive index with varying focus position. Given that the depth of field [DOF = $\lambda \, (n^2-\text {NA}^2)^{0.5} / (2 \, \text {NA}^{2})$] is less than 2 µm and the z-axis micrometer has 10 µm division in this study, the precision within ± 5 µm focus position is only 2 $\times$ 10$^{-4}$. Therefore, the $10^{-4}$ precision in our interferometry measurements, which is much smaller than the random uncertainties in previous studies on water from 0.01^13,14,18,19^ to 0.001^12^ and to 0.0005^12^, is reasonable. As our method provides the refractive index and thickness of a sample independently (see section “Refractive index of a compressed sample”), we obtain also a random error for the sample thickness measurement to be 0.01$\%$ from the same ten measurements described above.

### Image aberration

The use of thick diamond anvils as the optical windows, their misalignment and cupping^38^, and the high NA objective lens possibly cause the image aberration as stated in “Correction of image aberration”. Since the deformation of anvils is negligible in the few GPa range and the anvils are well aligned using a typical Newton ring method, the correction relation for the image aberration (Eq. 5) in the individual DACs would not change during the tests, but could be different from each other. To estimate the related uncertainty, we measure another empty DAC to obtain the correction relation $r'_{ab}$ and recalculate the refractive index of water in Fig. 6a with this relation. The difference between the initial and recalculated indices shows a standard deviation of 5.0 $\times$ 10$^{-4}$ as shown in Fig. 6c, which we consider as our dominant sources of systematic uncertainty, five times higher than the random uncertainty (10$^{-4}$ in section “Focus position”). Then, the total uncertainty, a quadrature sum of the random and systematic uncertainties, is 5.1 $\times$ 10$^{-4}$. Following the error analysis on the refractive index, the systematic error is estimated to be 0.2$\%$ yielding a 0.2$\%$ total uncertainty on the determination of the sample thickness.

## Conclusion

We demonstrated the accuracy of the refractive index measurement in the DAC using our advanced interferometer setup. The improvements in the experimental setup and the data analysis methodology enable us to achieve a $10^{-4}$ random error. Using this technique, the index at 532 nm and the dispersion over 500–700 nm of water are reported in the liquid and ice VI phases with increasing pressure up to 2.21 GPa. We describe the linear index-density relations in each phase with the modified Gladstone–Dale model and reveal the pressure-induced change in molecular polarizability with the Lorentz–Lorens model. Detailed studies of the pressure, density, and wavelength dependences of the refractive index of water and water-rich planetary ices mixtures as well as their high-pressure electronic structures will be described in upcoming publications.

## Supplementary Information

Supplementary Information 1.
