# Author Correction: Interferometric measurements of refractive index and dispersion at high pressure

**DOI:** 10.1038/s41598-021-91668-4

**Published:** 2021-06-14

**Authors:** Yong‑Jae Kim, Peter M. Celliers, Jon H. Eggert, Amy Lazicki, Marius Millot

**Affiliations:** Lawrence Livermore National Laboratory, Livermore, CA 94550 USA

Correction to: *Scientific Reports* https://doi.org/10.1038/s41598-021-84883-6, published online 10 March 2021

The original version of this Article contained errors in References 3, 14, 22, 31, 35, 38, 40, 42, and 47 which were incorrectly given as:

3. Gladstone, J. & Dale, T. X. I. V. Researches on the refraction, dispersion, and sensitiveness of liquids. *Philos. Trans. R. Soc. Lond.* **153**, 317 (1863).

14. Shimizu, H., Nabetani, T., Nishiba, T. & Sasaki, S. High-pressure elastic properties of the VI and VII phase of ice in dense H_2_O and D_2_O

22. van Straaten, J. & Silvera, I. F. Equation of state of solid molecular H_2_H2H2 and D2D2D_2_ at 5 K. *Phys. Rev. B* **37**, 1989 (1988).

31. Brygoo, S. *et al.* Analysis of laser shock experiments on precompressed samples using a quartz reference and application to warm dense hydrogen and helium. *J. Appl. Phys.* **118**, 1 (2015) (**arXiv:1510.03301**).

35. Pruteanu, C. G., Ackland, G. J., Poon, W. C. K. K. & Loveday, J. S. When immiscible becomes miscible-methane in water at high pressures. *Sci. Adv.* **3**, 1 (2017).

38. Aebischer, N. Calculs de profils dissym triques observables sur des figures d’interf rences en ondes multiples sph riques. *Nouvelle Revue d’Optique Appliquée* **2**, 351 (1971).

40. Dunaeva, A. N., Antsyshkin, D. V. & Kuskov, O. L. Phase diagram of H2O: Thermodynamic functions of the phase transitions of high-pressure ices. *Sol. Syst. Res.* **44**, 202 (2010).

42. Courchinoux, R. & Lalle, P. Dynamic properties of water: Sound velocity and refractive index. In *booktitle AIP Conference Proceedings*, 370 (AIP, 1996) pp. 61–64

47. Ge, X. & Lu, D. Molecular polarizability of water from local dielectric response theory. *Phys. Rev. B* **96**, 1 (2017).

The correct references are listed below:

3. Gladstone, J. & Dale, T. XIV Researches on the refraction, dispersion, and sensitiveness of liquids. *Philos. Trans. R. Soc. Lond.* **153**, 317 (1863).

14. Shimizu, H., Nabetani, T., Nishiba, T. & Sasaki, S. High-pressure elastic properties of the VI and VII phase of ice in dense H_2_O and D_2_O. *Phys, Rev. B* **53**, 6107 (1996).

22. van Straaten, J. & Silvera, I. F. Equation of state of solid molecular H_2_ and D_2_ at 5 K. *Phys. Rev. B* **37**, 1989 (1988).

31. Brygoo, S. *et al.* Analysis of laser shock experiments on precompressed samples using a quartz reference and application to warm dense hydrogen and helium. *J. Appl. Phys.* **118**, 195901 (2015).

35. Pruteanu, C. G., Ackland, G. J., Poon, W. C. K. & Loveday, J. S. When immiscible becomes miscible-methane in water at high pressures. *Sci. Adv.* **3**, e170024 (2017).

38. Aebischer, N. Calculs de profils dissymétriques observables sur des figures d’interférences en ondes multiples sphériques. *Nouvelle Revue d’Optique Appliquée* **2**, 351 (1971).

40. Dunaeva, A. N., Antsyshkin, D. V. & Kuskov, O. L. Phase diagram of H_2_O: Thermodynamic functions of the phase transitions of high-pressure ices. *Sol. Syst. Res.* **44**, 202 (2010).

42. Courchinoux, R. & Lalle, P. Dynamic properties of water: Sound velocity and refractive index. *AIP Conference Proceedings*, **370**, 61 (1996).

47. Ge, X. & Lu, D. Molecular polarizability of water from local dielectric response theory. *Phys. Rev. B* **96**, 075114 (2017).

The original Article has been corrected.

