# Supplementary material for: Interferometric measurements of refractive index and dispersion at high pressure
Source: Sci Rep. 2021 Mar 10;11:5610. doi: 10.1038/s41598-021-84883-6 (PMC7970932; doi:10.1038/s41598-021-84883-6)
Supplement: Supplementary file 1 — Supplementary Information 1. [file 41598_2021_84883_MOESM1_ESM.pdf]

Supplementary information

Interferometric measurements of refractive index and dispersion at high pressure

Yong-Jae Kim,[1,2] Peter M. Celliers,[1] Jon H. Eggert,[1] Amy Lazicki,[1] and Marius Millot[1]
[1] *Lawrence Livermore National Laboratory, Livermore, CA 94550, USA.*
[2] *kim100@llnl.gov*

S1. Dispersion-induced error

In the interference spectrum obtained with the white light, the interference order (k) at a specific wavenumber (ν) is simply estimated with the oscillation period ($\Delta\nu$); $k = \nu/\Delta\nu$. This relation is derived by comparing the interference orders at two adjacent ith and $(i+1)$th fringe minima ($k_{i+1} = k_i + 1$) when the dispersion of the refractive index is negligible ($n_{s,i+1} \sim n_{s,i}$), like in air:

$$\begin{aligned} k_{i+1} - k_i &= 2n_{s,i+1}t_s\nu_{i+1} - 2n_{s,i}t_s\nu_i \\ &= 2n_{s,i}t_s\Delta\nu = 1, \end{aligned} \tag{S1}$$

and,

$$k = 2n_st_s\nu = \frac{\nu}{\Delta\nu}. \tag{S2}$$

This relation, however, is no longer valid for a sample with a non-negligible dispersion ($n_{s,i+1} \nsim n_{s,i}$), like water. For the sake of simplicity, by assuming the dispersion with a linear slope [$\alpha = dn/d\nu = (n_{i+1} - n_i)/\Delta\nu$], Eqs. S1 and S2 can be rewritten as

$$\begin{aligned} k_{i+1} - k_i &= 2n_{s,i+1}t_s\nu_{i+1} - 2n_{s,i}t_s\nu_i \\ &= 2t_s\left[\left(n_{s,i} + \alpha\Delta\nu\right)\nu_{i+1} - n_{s,i}\nu_i\right] \\ &= 2n_{s,i}t_s\left(\nu_{i+1} + \frac{\nu_{i+1}}{n_{s,i}}\alpha\Delta\nu - \nu_i\right) \\ &= 2n_{s,i}t_s\Delta\nu\left(1 + \frac{\nu_i + \Delta\nu}{n_{s,i}}\alpha\right) = 1, \end{aligned} \tag{S3}$$

and,

$$\begin{aligned} k_{disp} &= 2n_st_s\nu \\ &= \frac{\nu}{\Delta\nu}\left(1 + \frac{\nu + \Delta\nu}{n_s}\alpha\right)^{-1} \\ &= k\left(1 + \frac{\nu + \Delta\nu}{n_s}\alpha\right)^{-1}, \end{aligned} \tag{S4}$$

where k_{disp} is the interference order of a sample with a non-negligible, linear dispersion. The dispersion slope α is positive for water in the wavenumber and pressure ranges examined in this study (Fig. 8b), indicating that the measurement of $\nu/\Delta\nu$ (Eq. S2) in the interference spectrum overestimates the interference order (i.e., $k > k_{disp}$).

We only use the integer part of the measured k, $[k]$ in Eq. 3, to count the ring orders in the monochromatic interference patterns. To achieve the same integer parts in k and k_{disp} and eliminate the dispersion-related error in the ring pattern analysis, their difference must be smaller than at most one;

$$\begin{aligned} k - k_{disp} &= k\left[1 - \left(1 + \frac{\nu + \Delta\nu}{n_s}\alpha\right)^{-1}\right] \\ &= k\left(\frac{\nu + \Delta\nu}{n_s}\alpha\right)\left(1 + \frac{\nu + \Delta\nu}{n_s}\alpha\right)^{-1} < 1. \end{aligned} \tag{S5}$$

Then, as usually $\Delta\nu \ll \nu$, the k satisfying Eq. S5 and the corresponding sample thickness t_s are calculated as

$$\begin{aligned} k &< \frac{n_s}{(\nu + \Delta\nu)\alpha} + 1 \approx \frac{n_s}{\nu\alpha} \\ t_s &= \frac{k}{2n_s\nu} < \frac{1}{2\nu^2\alpha}. \end{aligned} \tag{S6}$$

As an example for water, the upper limits of k and t_s to avoid the dispersion-induced error are calculated to be 143.48 and 33.48 μm, respectively; n_s is approximated as 1.5, ν is 0.001429 (=1/700) nm^{-1}, and $\alpha(= dn/d\nu)$ near this wavenumber is measured as 7.318 nm in average (Fig. 8b).

We note that, even though Eq. S6 is satisfied, the difference in the integer part of k and k_{disp} can be different; for example, when $k = 100.1$ and $k_{disp} = 99.9$, their difference is only 0.2 but their integer parts differ by 1. This situation can be practically avoided by using the sample much thinner than the upper limit and measuring the interferograms when the first ring in the laser interference pattern is big enough (*e.g.*, $k_L - [k_L] = 0.9$).

S2. Hilbert transform

To normalize the intensity of the zero-mean interference spectrum (I_{exp}) in wavenumber space (ν), an envelope (or instantaneous amplitude, I_{amp}) is obtained using the Hilbert transform (HT)[S1,S2]. For an experimental spectrum I_{exp}, its HT (I_{HT}) can be obtained through the improper and convolution integrals:

$$\begin{aligned} I_{HT}(\nu) &= \frac{1}{\pi}\int_{-\infty}^{\infty}\frac{I_{exp}(x)}{\nu - x}dx \\ &= \left(\frac{1}{\pi x}\right) * I_{exp}(x) \\ &= F^{-1}\left\{F\left(\frac{1}{\pi x}\right)\cdot F\left[I_{exp}(x)\right]\right\} \\ &= F^{-1}\left\{\left[-i\cdot \operatorname{sgn}(x)\right]\cdot F\left[I_{exp}(x)\right]\right\}. \end{aligned} \tag{S7}$$

After zero-padding, the convolution integral with the symbol $*$ is computed with the forward and reverse Fourier transforms (F and F^{-1}) and the signum function (sgn) which returns 1, 0, or -1 for $x{>}0$, $x = 0$, or $x{<}0$, respectively.

Physically, the HT shifts the fringe phase by $-\pi/2$ while the amplitude is left unchanged. We can simply express I_{exp} and I_{HT} as

$$I_{exp}(\nu) = I_{amp}(\nu)\cos\varphi, \tag{S8}$$

and,

$$\begin{aligned} I_{HT}(\nu) &= I_{amp}(\nu)\cos(\varphi - \pi/2) \\ &= I_{amp}(\nu)\sin\varphi, \end{aligned} \tag{S9}$$

where φ is the instantaneous phase. Then, the analytic spectrum (or the analytic signal, I_a, which is a complex signal whose imaginary part is the HT of the real part) can be summarized in the form of Euler's formula:

$$\begin{aligned} I_a(\nu) &= I_{exp}(\nu) + iI_{HT}(\nu) \\ &= I_{amp}(\nu)\cos\varphi + iI_{amp}(\nu)\sin\varphi \\ &= I_{amp}(\nu)e^{i\varphi}. \end{aligned} \tag{S10}$$

Finally, the envelope of fringe spectrum, $I_{amp}(\nu)$, can be determined as a positive magnitude of analytic spectrum, $|I_a|$,

$$\begin{aligned} I_{amp}(\nu) &= |I_a| \\ &= \sqrt{I_{exp}^2(\nu) + I_{HT}^2(\nu)} \\ &= \exp\left\{\operatorname{Re}\left[\ln I_a(\nu)\right]\right\}. \end{aligned} \tag{S11}$$

One can further extend the use of the HT to measure the instantaneous phase, $\varphi(\nu)$, and frequency, $f(\nu)$, in oscillation signal.

$$\begin{aligned}\varphi(\nu) &= \arctan\left[\frac{I_{HT}(\nu)}{I_{exp}(\nu)}\right] \\ &= \mathrm{Im}\left[\ln I_a(\nu)\right], \end{aligned} \tag{S12}$$

and,

$$\begin{aligned} f(\nu) &= \frac{\omega(\nu)}{2\pi} = \frac{\dot{\varphi}(\nu)}{2\pi} \\ &= \frac{I_{exp}(\nu)\dot{I}_{HT}(\nu) - \dot{I}_{exp}(\nu)I_{HT}(\nu)}{2\pi I_{amp}^2(\nu)} \\ &= \frac{1}{2\pi}\,\mathrm{Im}\left[\frac{\dot{I}_a(\nu)}{I_a(\nu)}\right], \end{aligned} \tag{S13}$$

where ω is the instantaneous angular frequency and the overdot indicates differentiation with respect to ν.

References

[S1]S. L. Hahn, *Hilbert transforms in signal processing* (Artech House, 1996) p. 442.

[S2]M. Feldman, “Hilbert transform in vibration analysis,” Mechanical Systems and Signal Processing **25**, 735–802 (2011).
